# An mHealth pilot designed to increase the reach of prevention of mother-to-child transmission of HIV (PMTCT) across the treatment cascade in a resource-constrained setting in Tanzania

**DOI:** 10.1371/journal.pone.0212305

**Published:** 2019-02-15

**Authors:** Deborah S. K. Thomas, Sheana Bull, Elias C. Nyanza, Karen Hampanda, Michael Liedtke, Sospatro E. Ngallaba

**Affiliations:** 1 Department of Geography & Environmental Sciences, University of Colorado Denver, Denver, Colorado, United States of America; 2 Department of Community and Behavioral Health, Colorado School of Public Health, University of Colorado Anschutz Medical Campus, Aurora, Colorado, United States of America; 3 School of Public Health, Catholic University of Health and Allied Sciences, Mwanza, United Republic of Tanzania; 4 Colorado School of Public Health, University of Colorado Anschutz Medical Campus, Aurora, Colorado, United States of America; Paediatric Centre of Excellence, ZAMBIA

## Abstract

**Background:**

Data collection and integrated reporting between the multiple health facilities for supporting more efficient care linkages is an indispensable element for prevention of mother-to-child transmission of HIV (PMTCT) by fostering continuity of patient care and improving the treatment cascade for HIV-infected pregnant women. mHealth potentially presents timely solutions to the data challenges related to efficient and effective care delivery in resource-constrained settings, particularly in low- and middle-income countries.

**Methods:**

This randomized controlled pilot study used stratified random sampling for the selection of seven intervention and seven control sites in Misungwi, Tanzania, a rural district in the northwestern region. Twenty-eight health workers at seven intervention health facilities used the Tanzania Health Information Technology (T-HIT) system during a 3-month period from February 23, 2015, through May 23, 2015, to capture antenatal, delivery, and postnatal patient visits.

**Results:**

T-HIT was designed for use on tablets with the goal to improve reporting, surveillance and monitoring of HIV rates and care delivery in the remote and rural settings. Health workers successfully recorded 2,453 visits. Of these, 1,594 were antenatal visits, 484 deliveries were recorded, and 375 were postnatal visits. Within the antenatal visits, 96% of women had a single visit (1474). Healthcare workers were unable to test 6.7% of women antenatally for HIV.

**Conclusion:**

The T-HIT pilot demonstrated the feasibility for implementing an mHealth integrated solution in a rural, low-resource setting that links tablet-based surveillance, health worker capacity-building and patient reminders into a single robust and responsive system. Although the implementation phase was only three months, the pilot generated evidence that T-HIT has potential for improving patient outcomes by providing more comprehensive, linked, and timely PMTCT care data at the individual and clinic levels.

## Background

In parallel with many sub-Saharan countries, Tanzania has implemented numerous programs aimed at combating HIV/AIDS, including voluntary counseling and testing (VCT), lifelong anti-retroviral therapy (ART), and prevention of mother-to-child transmission of HIV (PMTCT). Starting in 2013, Tanzania rolled out the World Health Organization’s (WHO) “Option B+” strategy, which recommends lifelong ART for all pregnant/postpartum women [1]. Despite significant gains in HIV testing and ART initiation among pregnant women, ongoing gaps in the continuity of HIV care hinder Tanzania from achieving the global goal of eliminating mother-to-child transmission [2, 3].

The national HIV prevalence in Tanzania is estimated at 4.5%, with heterosexual contact followed by mother-to-child transmission as the leading causes of HIV transmission in the country [4]. Although an estimated 14,000 infections were averted by PMTCT programs in Tanzania in 2017, 11,000 children were newly infected with HIV due to disconnects in access to quality care and high rates of loss to follow-up [4]. In 2017, 85% of pregnant women living with HIV in Tanzania had access at ART through PMTCT programs [4]. However, Cichowitz et al. found that in a sample of over 600 pregnant women initiating ART in Tanzania, over 50% were lost to follow-up in two years and over 19% were lost within the first month of antenatal care [2].

A lack of standardized surveillance and monitoring for access to, and delivery of, care exacerbates interruptions and delays in care continuity for PMTCT within the Tanzanian health system [5]. At present, no integrated surveillance or patient record keeping exists, resulting in poor follow-up with patients, as well as time delays between testing positive for HIV and accessing treatment and follow-up. This gives rise to significantly reduced care for women and infants, particularly in rural areas, and limits achieving “Option B+” goals. Data collection and integrated reporting between the multiple health facilities for patient monitoring and tracking is an indispensable element for ensuring continuity of patient care and improving the treatment cascade for HIV-infected pregnant women through more efficient linkages [5].

The growing number of cell phone and tablet-based strategies that facilitate decision-making for care providers, as well as health promotion and disease self-management for individuals, represents a digital health revolution [6]. These strategies are often referred to as “mHealth,” “eHealth” or digital Health, and have accompanied the explosive growth in cell phone ownership and use globally. With an estimated 4.4 billion mobile broadband subscriptions worldwide by the end of 2018, cell phone ownership is extensive in low and middle-income countries (LMICs) although a digital divide still exists [7], presenting tremendous opportunities for mHealth solutions to the challenges related to efficient and effective health care delivery in resource poor settings. Indeed, there is increasing recognition that mHealth approaches can be successfully leveraged to improve outcomes across the HIV care continuum [8, 9], including PMTCT [10]. This has lead the World Health Organization to advocate for the use of mHealth in promoting patient ART adherence [11].

Although most mHealth approaches, including those targeting HIV outcomes, are directed at individuals [8, 12], significant potential exists for using an integrated systems approach with healthcare workers, particularly in under-resourced settings like Tanzania [13]. For instance, a growing body of evidence suggests mHealth applications can facilitate health care delivery with tools that support health behavior and disease surveillance, curating medical records, decision aids for care providers, and communication with patients [14–16]. mHealth tools and programs that include logic models and the use of conceptual and/or theoretical frameworks may offer enhanced benefit and ultimately may contribute to improvements in population health when widely disseminated and taken to scale [8, 17].

While evidence of the benefits of mHealth decision aids for health promotion and care delivery is growing in rural and remote settings in sub-Saharan Africa, documentation of their efficacy is relatively limited, particularly when aimed at the health delivery system in resource-limited settings [18]. Most studies focus on text-based interventions for prevention and self-management for behavior change at the individual level [15,16]. Attention has broadened toward targeting healthcare workers in rural and resource-poor settings as a possible sustainable intervention model [12,17]. Findings demonstrate that mHealth can improve behavioral and disease surveillance, communication between healthcare workers and their patients, between healthcare workers and clinic staff, and between healthcare workers and their supervisors. When healthcare workers use mHealth tools, evidence shows an association with improved compliance with care delivery protocols, as well as improved patient outcomes [18].

The Tanzania Health Information Technology (T-HIT) tablet-based integrated mHealth system was developed and piloted as an intervention to support improved monitoring, linking, and communication of HIV rates along with pregnancy risk factors for informing PMTCT care delivery. This paper considers whether a mHealth solution can successfully be implemented in a rural, resource-limited setting and if results show evidence of improved capture and linkage of patient data across the continuum of care to inform patient care at the clinic and district levels. Specifically, this paper descriptively presents the data healthcare workers entered into T-HIT at the facility-level as an indication of feasibility to implement and capture HIV status of pregnant women across antenatal, delivery, postnatal visits.

## Study design and methods

This randomized controlled pilot study examined the implementation of the T-HIT system. T-HIT, which included electronic patient data collection, reporting, texting, decision-aids, was targeted to healthcare workers at seven facilities in Misungwi District, Tanzania with the goal of providing a proof-of-concept mHealth system-level solution for improving the HIV treatment cascade in a remote and rural settings in northwestern Tanzania.

### Study area

In Tanzania, the ward is the smallest geographic administrative unit (somewhat like a county in the U.S., though usually smaller in area). Wards are aggregated into districts, which are in turn aggregated into regions. The Tanzanian health system is organized in a pyramid structure with dispensaries being the smallest care provider, then health centres, district hospitals, and finally regional hospitals. Misungwi District, with a population estimate of 351,609 in 2012 [19], had 42 total health facilities: 36 dispensaries, 4 health centres, and 2 hospitals, one of these a District Hospital. The district was selected because of its rural setting, health care structure, and current paper data collection approach, which are reflective of many settings across sub-Saharan Africa.

The individual dispensaries, health centres, and hospital currently maintain hand-written individual records of antenatal visits, delivery and postnatal visits, along with HIV testing and/or treatment. At all facilities in the district, care delivered during a woman’s visit is documented in separate paper log books, depending on the type of visit and care received. There is a log for VCT, another log for antenatal, another for delivery information and another for postnatal care. Additionally, data from a visit are also entered onto the maternal and child health card that the woman maintains herself. There are no protocols in place that link logs to each other. Consequently, no centralized system documents all care that a woman receives, either at that facility or centrally.

Healthcare workers submit copies of the summary records to the district monthly where the numbers are aggregated to yield district-level data. Data are not systematically assessed at the sub-district level (ward or health facility), and district reporting has significant delays, sometimes months. The lack of electronic health records is typical across sub-Saharan Africa, where paper records are most common, limiting high quality data for decision-support [20, 21].

### System design

**Fig 1:**
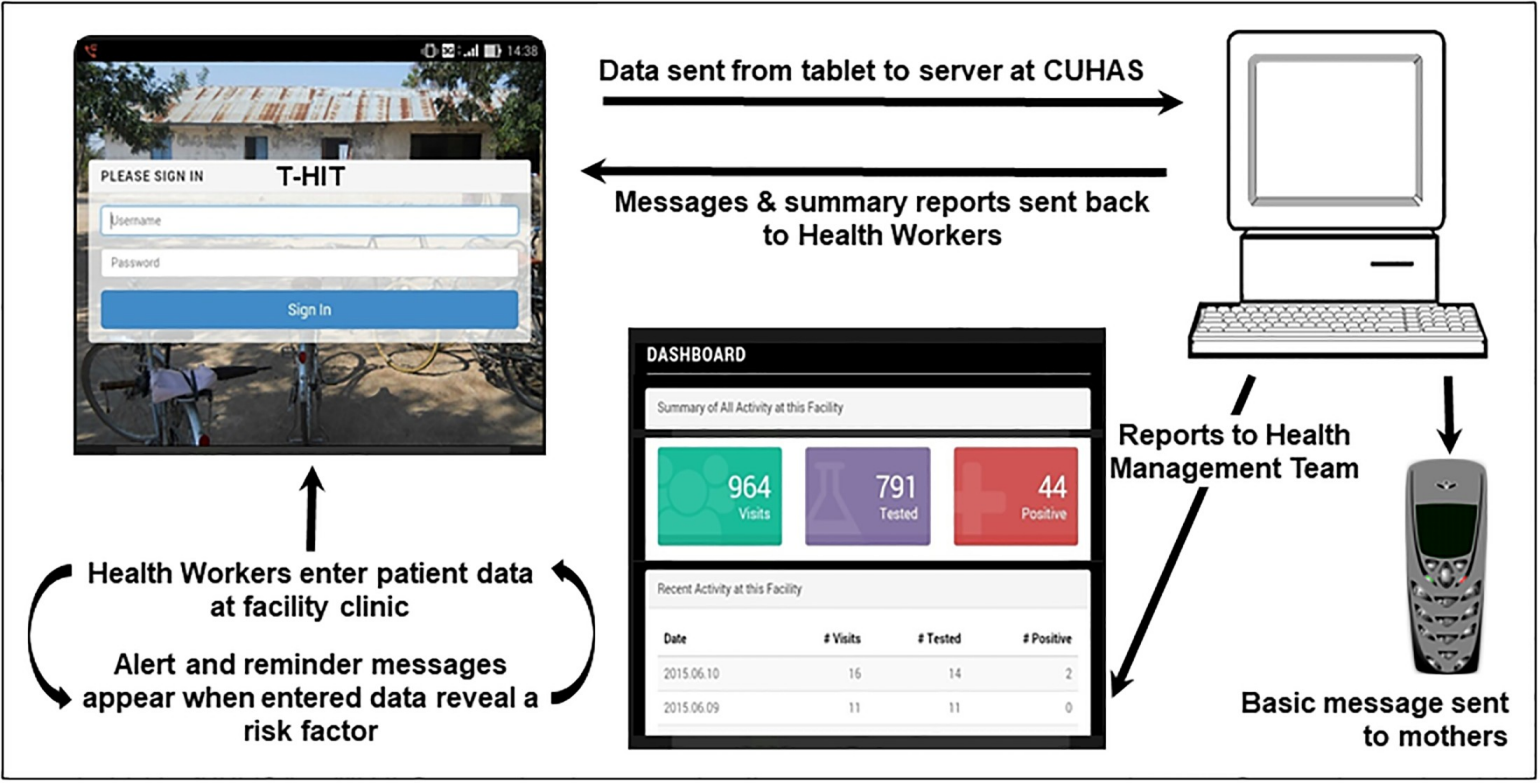
T-HIT system data flows.

The T-HIT system was designed to allow for synchronization of the disparate data. The T-HIT application programming interface (API) was developed for an Android tablet to capture patient data during antenatal, delivery, and postnatal visits, transferring encrypted data via the global system for mobile communications (GSM) network to a server at the Catholic University of Health and Allied Sciences in Mwanza, Tanzania. Fig 1 depicts the entire T-HIT system and data flow process, which includes documentation of testing for HIV, initiation and ongoing adherence to ART, and tests performed at each antenatal visit. Importantly, collected data elements mirrored data that are already collected on paper by healthcare workers related to PMTCT and antenatal care delivery and were captured through dropdown and selection options designed for simplicity. One slight variation was added that included an option for “unable to test” for HIV, rather than only yes or no. On paper records, healthcare workers document only if a woman is tested for HIV (yes or no), and protocols do not require further elucidation of why testing did not occur or if there was an inability.

In addition to the data collection function, the interface provided immediate feedback alerts if any pregnancy risk factors were identified and delivered weekly educational messages to healthcare workers about PMTCT. Both District Management Team Members and healthcare workers could observe the number of patients seen, the number tested for HIV and the number of HIV positive patients in near real-time (weekly) for that health facility and for all facilities combined on the dashboard upon logging into the system. In addition, a single text message was automatically sent seven days after the visit to mothers who agreed to provide a cell phone number and receive a message; it contained no health information and simply thanked them for their visit to the clinic. T-HIT provided summary reports to decision-makers at a facility or the district on a weekly and monthly basis, directly addressing the current limited information flows with current paper record-keeping.

### Site selection

**Fig 2:**
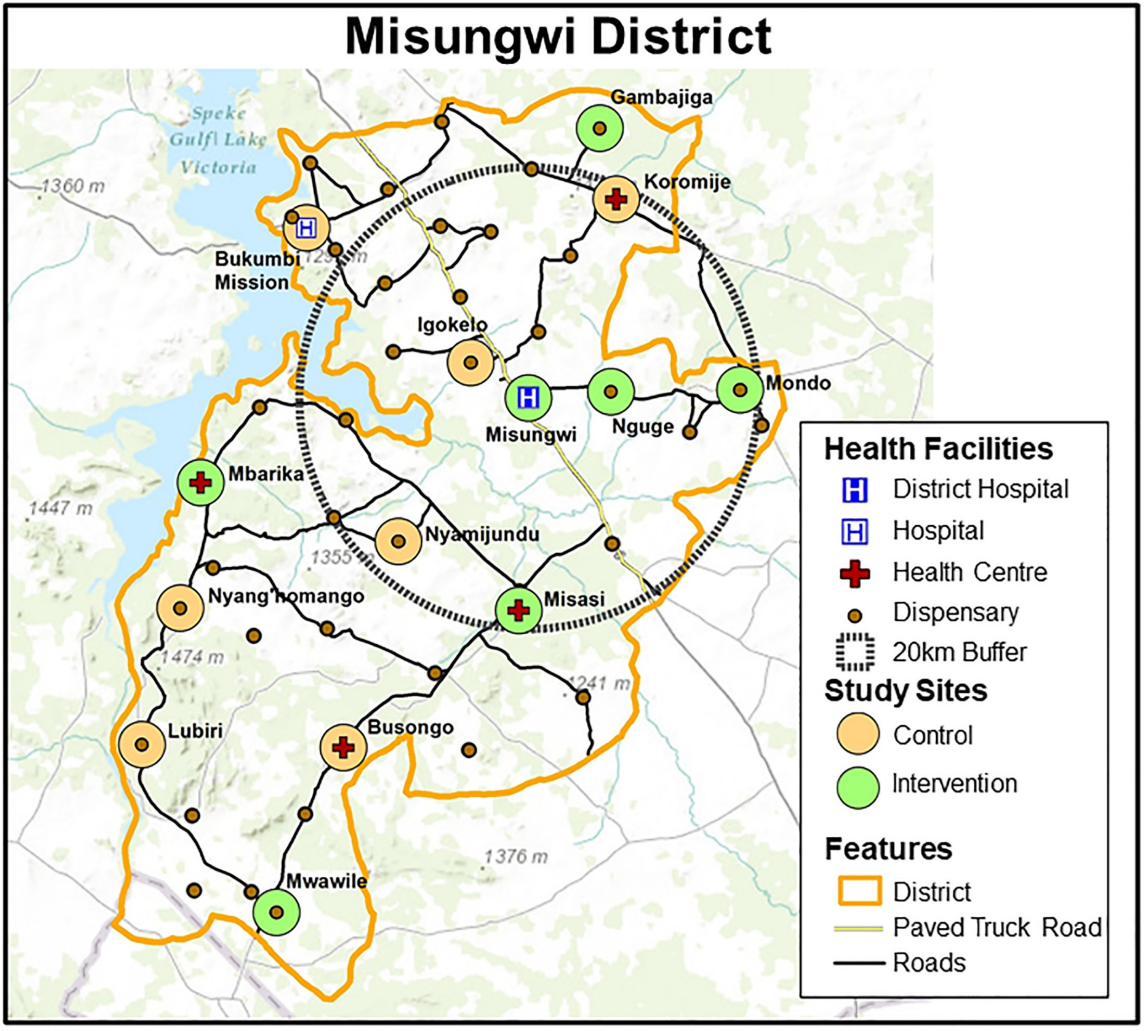
Misungwi district, Tanzania, health facilities and study sites.

Prior to the pilot data collection, an inventory of all facilities in the district was conducted in summer, 2014, and updated in January, 2015, to establish cell phone connectivity and availability of electricity, as well as to develop base maps of facilities and roads (Fig 2). Facility location and roads were collected with a global positioning system (GPS) and mapped using *ArcGIS version 10*.*2* [22] geographic information systems (GIS) software to guide the selection of the facilities.

The pilot study used stratified random sampling for the selection of seven intervention and seven control sites (Table 1), which were used in the full study [23]. Only the intervention sites are reported here to describe the activity recorded within the T-HIT system. The stratification was based on the level of the health facility in the health delivery system, along with distance from the district hospital (less or more than 20 kilometers) and experience with PMTCT (had PMTCT in 2010 or started later). Twenty-seven clinic-based healthcare workers were recruited across the seven intervention facilities (one hospital, two health centres, and four dispensaries), ranging from a minimum of three healthcare workers at three of the dispensaries, four healthcare workers at one dispensary/one health centre, to five healthcare workers at one health centre and the District Hospital.

**Table 1:** T-HIT intervention and control sites.

*Intervention Sites*	*Control Sites*
**Misungwi District Hospital**	**Bukumbi Mission Hospital**
Health Centre (close): **Misasi**	Health Centre (close): **Koromije**
Health Centre (far): **Mbarika**	Health Centre (far): **Busongo**
Dispensary (close/high experience): **Mondo**	Dispensary (close/high experience): **Igokelo**
Dispensary (close/low experience): **Nguge**	Dispensary (close/low experience): **Nyamijundu**
Dispensary (far/high experience): **Mwawile**	Dispensary (far/high experience): **Lubiri**
Dispensary (far/low experience): **Gambajiga**	Dispensary (far/low experience): **Nyang’homango**

### Data collection

The research team provided participating healthcare workers with rigorous training for one week prior to the official implementation of the T-HIT system. Healthcare workers entered data into the system using their unique T-HIT login from February 23, 2015 through May 23, 2015, a period long enough to demonstrate feasibility and potential for adoption. Tablets were distributed to each of the facilities for data entry, rather than to a healthcare worker. Each patient obtained a unique identification number derived from a combination of height, weight, and initials of parents to protect privacy and confidentiality, but at the same time could be tracked through the system.

Healthcare workers continued to enter data on paper at the intervention sites for the duration of this pilot study to ensure standard of care was being met. Log book paper records were monitored at both the control sites and intervention sites to provide a mechanism for evaluating the data entered into the T-HIT system. Comparisons between the paper records (intervention and control) and the electronic data (intervention only) are detailed elsewhere [23] and show that the data collected through T-HIT was more complete than paper logs in documenting antenatal visits, HIV tests and HIV positive results. This assessment also demonstrated errors in data entry in the T-HIT system in one of the intervention sites that were the result of imperfect system adoption, underscoring the importance of regular system monitoring to reduce data entry errors, a best practice regardless of data entry modality (i.e. paper or electronic system).

### Analysis

All data from the T-HIT system collected during the pilot period were included for analysis. Data were aggregated and reported at the health facility level to maintain healthcare worker confidentiality and because the goal was to assess a system-level intervention aimed at healthcare workers, not to evaluate individual work quality or quantity. Descriptive statistics were used to document frequencies of overall visits in T-HIT sites and percentages of women in each site who were screened for HIV in order to establish that health workers can successfully enter digital data and that the system could support sending data and information in a resource limited environment.

### Ethical, consent, and permissions

Ethical approval was obtained from the Conjoint Catholic University of Health and Allied Sciences and Bugando Medical Centre Research Review and Ethics Committee (Ref. *CREC/051/2013*), the Tanzania National Institute for Medical Research (*NIMR/HQ/R*.*8a/Vol*.*IX/1662*), and the Colorado Multiple Institutional Review Board (Protocol 13–2166). Permission to conduct research in in Tanzania was also obtained from the Tanzania Commission for Science and Technology (COSTECH) and Regional and District authorities in Mwanza and Misungwi respectively. Healthcare worker participation in the study was voluntary, and all who were approached agreed to take part. The need to consent was waived by the ethics committees for the inclusion of patient records.

## Results

During the intervention period, 27 healthcare workers used T-HIT at seven health facilities to document 2,453 visits (Table 2). Of these, 1,594 were antenatal visits, 484 deliveries were recorded over the pilot period, and 375 were postnatal visits. Even in the relatively short study period, 56 women had more than one visit across visit types, 48 had two visits, seven had three, and only one woman had four visits. Of the 56 women with more than one antenatal visit, 12 recorded a delivery and seven registered a postnatal visit. T-HIT captured six women with more than one antenatal visit with the delivery and postnatal visit.

**Table 2:** T-HIT intervention phase electronic records for clinic visits.

	Misungwi District Hospital Total N, Mean, STD	Misasi Health Centre Total N, Mean, STD	Mwawile Dispensary Total N, Mean, STD	Mbarika Health Centre Total N, Mean, STD	Mondo Dispensary Total N, Mean, STD	Nguge Dispensary Total N, Mean, STD	Gambajiga Dispensary Total N, Mean, STD	Total Total N, Mean, STD
**ANTENATAL VISITS**								
Mean number of antenatal visits by week	558 40.6 +/- 15.6	69 4.9 +/- 5.5	187 13.4 +/- 8.8	226 16.1 +/- 11.0	205 14.6 +/- 8.8	175 12.5 +/- 8.1	164 11.7 +/- 6.2	1594 16.27 +/- 3.38
Mean number of women tested for HIV at antenatal care by week	193 14.1 +/- 11.0	6 0.46 +/- 1.13	97 6.9 +/- 5.2	121 9.0 +/- 6.7	39 2.5 +/- 3.6	139 10.0 +/- 7.8	100 7.7 +/- 4.1	695 7.2 +/- 3.2
Mean number of women unable to test for HIV at antenatal care by week	23 1.6 +/- 3.5	* 0.2 +/- 0.4	24 1.7 +/- 3.6	9 0.6 +/- 1.2	34 2.4 +/- 2.7	10 0.7 +/- 2.2	0 0 +/- 0	103 1.1 +/- 1.4
Mean number of women testing positive for HIV at antenatal care by week	26 1.9 +/- 1.3	5 0.4 +/- 0.6	10 0.7 +/- 1.4	5 0.4 +/- 0.8	0 0.0 +/- 0.0	6 0.4 +/- 0.5	* 0.2 +/- 0.6	55 0.6 +/- 0.5
**DELIVERY VISITS**								
Mean number of delivery visits by week	181 12.9 +/- 9.5	112 8.0 +/- 5.8	62 4.4 +/- 3.6	57 4.1 +/- 2.9	0 0 +/- 0	26 1.9 +/- 3.7	46 3.3 +/- 1.9	484 4.9 +/- 3.0
Mean number of women tested for HIV at delivery by week	6 0.4 +/- 1.1	7 0.5 +/- 1.2	21 1.5 +/- 1.7	27 1.9 +/- 2.0	0 0 +/- 0	23 1.6 +/- 3.7	31 2.2 +/- 2.3	115 1.2 +/- 1.5
Mean number of women unable to test for HIV at delivery by week	* 0.3 +/- 0.6	* +/- 0.4	12 0.9 +/- 1.8	* +/- 0.5	0 0 +/- 0	* +/- 0.8	0 0 +/- 0	23 0.2 +/- 0.06
Mean number of women testing positive for HIV at delivery by week	* 0.1 +/- 0.3	* 0.3 +/- 1.1	* 0.3 +/- 0.8	* 0.3 +/- 0.6	0 0 +/- 0	0 0 +/- 0	* 0.1 +/- 0.4	15 0.2 +/- 0.4
**POSTNATAL VISITS**								
Mean number of postnatal visits by week	23 1.6 +/- 2.2	59 4.2 +/- 5.3	* 0.1 +/- 0.4	11 0.8 +/- 1.2	83 5.9 +/- 4.5	111 7.9 +/- 5.4	86 6.1 +/- 2.6	375 3.8 +/- 2.0
Mean number of women tested for HIV at postnatal visit by week	* 0.1 +/- 0.3	0 0 +/- 0	0 0 +/- 0	* 0.1 +/- 0.3	* 0.2 +/- 0.4	92 6.6 +/- 5.2	58 4.1 +/- 2.6	155 1.6 +/- 2.0
Mean number of women unable to test for HIV at postnatal visit by week	0 0 +/- 0	1 0.1 +/- 0.3	1 0.1 +/- 0.3	0 0 +/- 0	5 0.4 +/- 0.8	4 0.3 +/- 1.1	0 0 +/- 0	11 0.11 +/- 0,4
Mean number of women testing positive for HIV at postnatal visit by week	* 0.3 +/- 0.6	* 0.1 +/- 0.3	0 0 +/- 0	0 0 +/- 0	0 0 +/- 0	0 0 +/- 0	0 0 +/- 0	5 0.1 +/- 0.2

(*) Less than 5 women recorded

Within the antenatal visits, 96% of women had a single visit (1,474). Over one-third of the antenatal visits (35%, N = 556) were at the District Hospital. There were between 69 and 223 antenatal visits to dispensaries and health centres, with the highest average number of visits in Misungwi District Hospital (mean = 40.6 visits) and between 4.9 and 16.1 weekly visits in the health centres and dispensaries. We documented outcomes from 695 HIV tests that took place during an antenatal visit in the pilot period; of these, 55 women tested positive (3.5% of the 1530 unique women seen during the pilot). Importantly, the T-HIT system included an option for “unable to test” for HIV, likely due to a lack of testing materials at the clinic. Healthcare workers documented 103 women unable to test antenatally (6.7% of the unique women seen).

Of 484 deliveries recorded, 363 were in the hospital, although the number home births recorded is likely low due to a relatively low number of postnatal visits recorded in the system. Healthcare workers using T-HIT documented delivery HIV testing outcomes among 447 women. Of these, there were 15 women testing positive (3.3% of those tested at delivery); as with antenatal visits, we also documented that 23 women were unable to test at delivery. Finally, women also had the opportunity to test at a postnatal visit. There were 155 women of the 375 postnatal visits recorded in T-HIT where outcomes of HIV testing were documented; of these, 5 were positive (1.7%) and 11 (3%) were unable to test.

The T-HIT system documented few cases of syphilis overall, and elucidated challenges in testing for TB, where TB status was available for 1175 of the 1530 unique women. During the pilot, 130 women were tested for TB at an antenatal visit, ranging from 0 to 36, for an average of 1.4 women each week. T-HIT also documented that there were 406 women (26%) with one or more risk factors for poor pregnancy outcomes, including hypertension, being HIV infected, having a prior C-Section, anemia, short stature, more than five previous pregnancies or advanced age. Most women with a risk factor had only one. The highest proportions with one or more risk factors are seen in the Misasi Health Centre (44.9%), and the lowest in Nguge Dispensary (14.3%).

## Discussion

Twenty-seven health workers successfully entered antenatal, delivery, and postnatal data during the pilot, documenting challenges in testing for HIV and TB and noting pregnancy risk. The fact that these data were transmitted almost immediately during the pilot period from the study sites to the central hospital, providing brief summaries in near real-time, is of critical importance. Standard of care captures hand-written data on paper that are aggregated in monthly reports and sent to the district hospital, where they are then aggregated with other facility data to produce district reports submitted to the regional administrator. This represents a significant delay for decision-making. As such, implementation of the T-HIT system provided an improvement on access to data summaries that could be evaluated by the facility or at the district level within a week. Further, an individual health facility typically does not conduct data analysis, nor are the district-level reports disaggregated at the facility level. Thus, the ability for an individual facility to observe near real-time summaries of activity is an advancement in information sharing. Additionally, the reports provide a new opportunity to compare health facility activity at the sub-district level.

Notably, standard of care of paper data recording for HIV testing does not currently capture the inability to perform an HIV test, only that the HIV test result was yes or no. T-HIT did capture this relevant piece of data. When healthcare workers were asked about these data, they conveyed this is frequently because testing materials were unavailable. This is perhaps a critical contribution that T-HIT offered during the pilot, as this was not previously systematically captured in data collection. Thus, T-HIT identified important challenges in adherence to the Option B+ protocol with high rates of inability to test documented at several facilities, for example, Mwawile (13.6%) and Mondo (19%). Of interest, Mwawile also has the highest HIV incidence rate among the health facilities in the study. The inability to test is particularly challenging for PMTCT since many women are not likely to return for subsequent antenatal visits. The rapid transfer of this information could offer immediate actionable opportunities to restock testing materials at a facility and to reinforce the need for a mother to return for a follow-up visit.

The T-HIT system exhibited the potential for linking individual patient visits across time using a unique identifier. T-HIT recorded 56 women who attended more than one visit during the study period, signifying the promise of the system for linking currently disconnected data. Although the 3-month study period was short, the system could document multiple antenatal visits and followed women across visit type to delivery and postnatal visits. The women who were enrolled towards the beginning of the study could potentially have repeat antenatal, delivery, or postnatal visits recorded. Connection of visit types would likely improve over a longer period of implementation time. Additionally, using the individual patient’s Reproductive and Child Health Clinic Number, rather than a uniquely generated identifier that was somewhat cumbersomely used for the T-HIT system, would likely improve linkages. Importantly, T-HIT captured all maternal health-related data at each visit, offering the possibility for monitoring a woman’s health.

The three-month implementation success suggests the T-HIT system also has the potential for closer monitoring of ART adherence for mothers, better advanced planning for hospital delivery when mothers are HIV-infected, and opportunities for improvements in adherence to medication for newborns. These factors offer evidence that the T-HIT system has potential to improve the treatment cascade for HIV infected mothers and their babies. Finally, the system also allows for advantages that go beyond PMTCT. With timely transmission of information about high-risk pregnancies, for example, districts can be more proactive about monitoring these pregnancies and planning for hospital deliveries when appropriate, thereby increasing relevance for HIV prevention, and at the same time improving maternal and child outcomes.

While this pilot and other research suggests many benefits from employing mHealth solutions for healthcare workers, potential downfalls with relying on mHealth persist as well. The use of mHealth tools is not universal, and we know little about whether and how to sustain use among healthcare workers over time. Ongoing use will require buy-in from policy makers and directors within care delivery systems, high quality, ongoing training and motivational tools to incentivize sustained use [18].

## Conclusion

This paper demonstrated the feasibility for implementing an mHealth integrated solution in a rural, low-resource setting that links tablet-based surveillance, health worker capacity-building and patient reminders into a single robust and responsive system. Healthcare workers could enroll women in a rural setting using T-HIT and could document important clinical data relevant to PMTCT. The mHealth system successfully allowed for the weekly and monthly compilation of data, comparison of health facility PMTCT activity, and the potential linking of patient data across visits and care delivery. Although the implementation phase was only three months, the pilot generated evidence that a tablet-based system can successfully record and deliver critical PMTCT data in a resource-limited environment. This system allows for real-time understanding of perinatal care in remote settings and offers the potential for care delivery systems to respond much more quickly to needs for HIV testing materials and antiretroviral medication than is currently afforded. Taken together, the T-HIT pilot provides evidence that an mHealth integrated system could contribute to reducing the HIV treatment cascade and ultimately improve patient outcomes.

## References

[1] UNAIDS, Global Plan Towards the Elimination of New HIV Infections Among Children by 2015 and Keeping their Mothers Alive, in Countdown to Zero. 2011, Joint United Nations Programme on HIV/AIDS (UNAIDS).

[2] Cichowitz C, Mazuguni F, Minja L, Antelman G, Ngocho J, Knetel BA et al, Vulnerable at Each Step in the PMTCT Care Cascade: High Loss to Follow Up During Pregnancy and the Postpartum Period in Tanzania. AIDS Behav, 2018. 10.1007/s10461-018-2298-8 PMC6467690 30327997

[3] (TACAIDS), T.C.f.A. Tanzania third national multi-sectoral strategic framework for HIV and AIDS (2013/14-2017/18).. 2013 28 December 2018]; http://www.nationalplanningcycles.org/sites/default/files/country_docs/Tanzania/nmsf-iii_eng_final_report_2013mail.pdf.

[4] UNAIDS. Country FactSheet: United Republic of Tanzania: Data. 2018 [cited 2018; http://www.unaids.org/en/regionscountries/countries/unitedrepublicoftanzania.

[5] Knettel BA, Cichowitz C, Ngocho JS, Knipler ET, Chumba LN, Mmbaga BT, et al, Retention in HIV Care During Pregnancy and the Postpartum Period in the Option B+ Era: Systematic Review and Meta-Analysis of Studies in Africa. J Acquir Immune Defic Syndr, 2018 77(5): p. 427–438. 10.1097/QAI.0000000000001616 29287029 PMC5844830

[6] de Jongh T, Guroi-Urganci I, Vodopivec-Jamsek V, Car J, and Atun R, Mobile phone messaging for facilitating self-management of long-term illnesses. Cochrane Database Syst Rev, 2012 12: p. CD007459 10.1002/14651858.CD007459.pub2 23235644 PMC6486189

[7] (ITU)., I.T.U. Measuring the Information Society Report. 2018]; http://www.itu.int/pub/D-IND-ICTOI-2018

[8] Kemp CG and Velloza J, Implementation of eHealth Interventions Across the HIV Care Cascade: a Review of Recent Research. Curr HIV/AIDS Rep, 2018 15(6): p. 403–413. 10.1007/s11904-018-0415-y 30171519 PMC6289676

[9] Henny KD, Wilkes AL, McDonald CM, and Neumann MS, A Rapid Review of eHealth Interventions Addressing the Continuum of HIV Care (2007–2017). AIDS Behav, 2018 22(1): p. 43–63. 10.1007/s10461-017-1923-2 28983684 PMC5760442

[10] Odeny TA, Bukusi EA, Cohen CR, Yuhas K, Camlin CS, and McClelland RS, Texting improves testing: a randomized trial of two-way SMS to increase postpartum prevention of mother-to-child transmission retention and infant HIV testing. AIDS, 2014 28(15): p. 2307–12. 10.1097/QAD.0000000000000409 25313586 PMC4834137

[11] WHO, Consolidated guidelines on the use of antiretroviral drugs for treating and preventing HIV infection: Recommendations for a public health approach. 2013, World Health Organization: ACW, London. 24716260

[12] Odeny TA, Newman M, Bukusi EA, McClelland RS, Cohen CR, and Camlin CS, Developing content for a mHealth intervention to promote postpartum retention in prevention of mother-to-child HIV transmission programs and early infant diagnosis of HIV: a qualitative study. PLoS One, 2014 9(9): p. e106383 10.1371/journal.pone.0106383 25181408 PMC4152282

[13] White A, Thomas DS, Ezeanochie N, and Bull S, Health Worker mHealth Utilization: A Systematic Review. Comput Inform Nurs, 2016 34(5): p. 206–13. 10.1097/CIN.0000000000000231 26955009 PMC4860109

[14] Pop-Eleches C, Thirumurthy H, Habyarimana JP, Zivin JG, Goldstein MP, deWalque D, et al, Mobile phone technologies improve adherence to antiretroviral treatment in a resource-limited setting: a randomized controlled trial of text message reminders. AIDS, 2011 25(6): p. 825–34. 10.1097/QAD.0b013e32834380c1 21252632 PMC3718389

[15] Prue CS, Shannon KL, Khyang J, Edwards LJ, Ahmed S, Ram M, et al, Mobile phones improve case detection and management of malaria in rural Bangladesh. Malar J, 2013 12: p. 48 10.1186/1475-2875-12-48 23374585 PMC3585886

[16] Lund S, Hemed M, Nielsen BB, Said A, Said K, Makungu MH, et al, Mobile phones as a health communication tool to improve skilled attendance at delivery in Zanzibar: a cluster-randomised controlled trial. BJOG, 2012 119(10): p. 1256–64. 10.1111/j.1471-0528.2012.03413.x 22805598

[17] Källander K, Tibenderana JK, Akpogheneta OJ, Strachan DL, Hill Z, ten Asbroek AH, et al, Mobile health (mHealth) approaches and lessons for increased performance and retention of community health workers in low- and middle-income countries: a review. J Med Internet Res, 2013 15(1): p. e17 10.2196/jmir.2130 23353680 PMC3636306

[18] Braun R., Catalani C, Wimbush J, and Israelski D, (2013). Community health workers and mobile technology: a systematic review of the literature, PLoS. ONE, C. 10.1371/journal.pone.0065772. Accessed August 15, 2017. PMC3680423 23776544

[19] Tanzania National Bureau of Statistics. "Census 2012". Archived from the original on 5 March 2016. Accessed December 9, 2016.

[20] Odekunle FF, Odekunle RO, and Shankar S, (2017). Why sub-Saharan Africa lags in electronic health record adoption and possible strategies to increase its adoption in this region. International Journal of Health Sciences, 11(4): 59–64. 29085270 PMC5654179

[21] Williams F, Boren SA. The role of the electronic medical record (EMR) in care delivery development in developing countries: A systematic review. Inform Prim Care 2008;16:139–45. 18713530 10.14236/jhi.v16i2.685 PMC12392155

[22] ArcGIS [software program]. Redlands CA: Esri; 2015.

[23] Bull S, Thomas DSK, Nyanza EC, and Ngallaba SE. (2018) Tanzania Health Information Technology System (T-HIT): Results from a pilot test of a tablet-based system to improve prevention of mother-to-child transmission of HIV (PMTCT). JMIR mHealth and uHealth. 6 e16 10.2196/mhealth.8513. 10.2196/mhealth.8513 29335236 PMC5789159

